# Molecular Evolutionary Analyses of *Shiga toxin type 2 subunit A* Gene in the Enterohemorrhagic *Escherichia coli* (EHEC)

**DOI:** 10.3390/microorganisms12091812

**Published:** 2024-09-02

**Authors:** Ryusuke Kimura, Hirokazu Kimura, Tatsuya Shirai, Yuriko Hayashi, Yuka Sato-Fujimoto, Wataru Kamitani, Akihide Ryo, Haruyoshi Tomita

**Affiliations:** 1 Department of Bacteriology, Graduate School of Medicine, Gunma University, Maebashi-shi 371-8511, Gunma, Japan; m2220015@gunma-u.ac.jp (R.K.); tomitaha@gunma-u.ac.jp (H.T.); 2 Advanced Medical Science Research Center, Gunma Paz University, Takasaki-shi 370-0006, Gunma, Japan; shirai@niid.go.jp (T.S.); hayashi@paz.ac.jp (Y.H.); 3 Department of Health Science, Gunma Paz University Graduate School of Health Sciences, Takasaki-shi 370-0006, Gunma, Japan; 4 Department of Virology III, Infectious Disease Surveillance Center, National Institute of Infectious Diseases, Tokyo 208-0011, Japan; aryo@niid.go.jp; 5 Faculty of Healthcare, Tokyo Healthcare University, Tokyo 141-8648, Japan; y-fujimoto@thcu.ac.jp; 6 Department of Infectious Diseases and Host Defense, Gunma University Graduate School of Medicine, Maebashi-shi 371-8511, Gunma, Japan; wakamita@gunma-u.ac.jp

**Keywords:** enterohemorrhagic *Escherichia coli* (EHEC), *Shiga toxin type 2 subunit A* gene (*stx2A* gene), Shiga toxin type 2 (Stx2), molecular evolution, conformational epitopes, immunogenicity/pathogenicity

## Abstract

To better understand the molecular genetics of the *Shiga toxin type 2 subunit A* gene (*stx2A* gene), we collected many subtypes of *stx2A* genes and performed detailed molecular evolutionary analyses of the gene. To achieve the aim of the study, we used several bioinformatics technologies, including time-scaled phylogenetic analyses, phylogenetic distance analyses, phylodynamics analyses, selective pressure analyses, and conformational epitope analyses. A time-scaled phylogeny showed that the common ancestor of the *stx2A* gene dated back to around 18,600 years ago. After that, the gene diverged into two major lineages (Lineage 1 and 2). Lineage 1 comprised the *stx2a–2d* subtypes, while Lineage 2 comprised the *stx2e*, *2g*, *2h*, and *2o* subtypes. The evolutionary rates of the genes were relatively fast. Phylogenetic distances showed that the Lineage 2 strains had a wider genetic divergence than Lineage 1. Phylodynamics also indicated that the population size of the *stx2A* gene increased after the 1930s and spread globally. Moreover, negative selection sites were identified in the Stx2A proteins, and these sites were diffusely distributed throughout the protein. Two negative selection sites were located adjacent to an active site of the common Stx2A protein. Many conformational epitopes were also estimated in these proteins, while no conformational epitope was found adjacent to the active site. The results suggest that the *stx2A* gene has uniquely evolved and diverged over an extremely long time, resulting in many subtypes. The dominance of the strains belonging to Lineage 1 suggests that differences in virulence may be involved in the prosperity of the offspring. Furthermore, some subtypes of Stx2A proteins may be able to induce effective neutralizing antibodies against the proteins in humans.

## 1. Introduction

Enterohemorrhagic *Escherichia coli* (EHEC) is thought to be an emerging and zoonotic pathogen that first appeared in the 1980s [1]. Currently, EHEC has been detected worldwide, and the World Health Organization estimates that over 200 million people per year may become infected with this pathogen [2]. Moreover, EHEC infection not only causes dysentery-like acute gastroenteritis but may also lead to serious symptoms such as hemolytic uremic syndrome (HUS) [2]. Over 5% of apparent infection cases may result in HUS, and around 3% of HUS cases may be lethal [3]. Therefore, EHEC infection is an important and serious disease burden in humans [1,2,3].

The main virulence factor of EHEC is the Shiga toxins (Stxs) [4]. Stxs are exotoxins classified into two types—Stx type 1 (Stx1) and Stx2 [4]. Previous reports have shown that some EHEC strains produce Stx1 alone, Stx2 alone, and both Stx1 and Stx2 [5,6]. Previous data have also shown that Stx1 derived from *E. coli* is essentially identical to Stx1 derived from *Shigella dysenteriae* [4]. While the molecular mechanisms of both Stx1 and Stx2 may be similar, Stx2 is considered to be more virulent than Stx1 [7]. Moreover, the amino acid sequences and structures between Stx1 and Stx2 are distinct [8,9]. Stx2 also has wide genetic and amino acid sequence divergences [8,10].

Previous reports have suggested some biological advantages of the Stx productions from *E. coli* [11,12]. For example, Stx may suppress the host’s immune system, resulting in *E. coli* being able to evade immune responses and persist in the host [13]. Moreover, Stx can causes severe diarrhea, facilitating transmission to other hosts through fecal matter [13]. Consequently, *E. coli* strains with the Stx can more effectively spread infections [12]. However, the relationship between the genetic divergences and the pathogenesis of Stx2 may not be exactly known [6,14].

The Stx2 protein consists of two subunits—Stx2A (approximately 33 kDa) and Stx2B (approximately 7.6 kDa) [12,15]. It has been suggested that a monomer of the Stx2A subunit protein (Stx2A) combines by non-covalent bonding with the Stx2B pentamer [12]. Previous reports have shown that Stx2A has a catalytic function as an RNA-N-glycosidase, and the enzyme leads to the inactivation of intracellular 60S ribosomes, resulting in the inhibition of various intracellular protein syntheses [16,17]. These actions may be responsible for the main pathogenesis of Stx2 protein [17,18]. Another subunit, the Stx2B can bind to a receptor expressing as globotriaosylceramide 3 (Gb3) on various epithelial cells in our body [17,19].

Given these contexts, to better understand molecular genetics, molecular evolutionary analyses in microbiology enable us to estimate various unknown issues, including molecular phylogenetics, phylodynamics, molecular structures, and functions of target molecules [20,21,22]. These data may also be able to elucidate the pathogenesis of various pathogens, including EHEC. The aim of this study is to elucidate the molecular function/evolution of the Stx2A and to detail estimate molecular structure and functions using bioinformatics technologies.

## 2. Materials and Methods 

### 2.1. Strains Used in This Study

Previous reports have suggested that the *stx2A* genes are classified into *stx2a*–*stx2o* based on their nucleotide sequence [23]. Thus, to analyze the molecular evolution of Stx2A, the full length of the *stx2A* genes were downloaded from GenBank (https://www.ncbi.nlm.nih.gov/genbank/; last accessed on 4 January 2024). We collected 916 strains and reference strains for each subtype (*stx2a*: AP026739, *stx2b*: AF043627, *stx2c*: AB015057, *stx2d*: DQ059012, *stx2e*: AJ567998, *stx2f*: CP039404, *stx2g*: KF932378, *stx2h*: AB048227, *stx2o*: MZ229604). Reference strains were selected based on EHEC testing and the diagnostic manual published by the national institute of infectious disease (NIID). For reference strains of unknown collection dates (*stx2f* and *2g*), we performed a Standard Nucleotide BLAST, and strains with high nucleotide sequence identity were substituted. Strains with missing data regarding the years or regions of isolation were excluded. We also excluded strains with identical DNA sequences (100% identity) determined using Clustal Omega (https://www.ebi.ac.uk/jdispatcher/msa/clustalo; accessed on 10 January 2024) [24]. Finally, we performed molecular evolutionary analysis on the 125 strains, including each subtype reference strain. Details of the present strains are shown in Supplementary Table S1.

### 2.2. Time-Scaled Phylogenetic Analyses

To evaluate the molecular evolution of the present strains, we constructed a phylogenetic tree using the Bayesian Markov chain Monte Carlo (MCMC) method in the BEAST 2 package (v2.6.7) [25]. First, we applied jModelTest2 to estimate the appropriate clock model, and HKY-Γ-I was selected. Next, the path-sampling/stepping-stone sampling marginal likelihood estimation method was used to select the best of the four clock models (strict clock, relaxed clock exponential, relaxed clock log normal, and random local clock) and the two prior tree models (coalescent constant population and coalescent exponential population). These were performed independently for all strains, and relaxed clock exponential and coalescent constant population were selected for the molecular evolutionary analysis. Effective sample size (ESS) was estimated using Tracer (v.1.7.2), and the ESS was confirmed to be greater than 200 for all parameters [26]. In addition, after a 10% burn-in, the phylogenetic tree was generated using TreeAnnotator (v2.6.7) and illustrated using FigTree (v1.4.0). Evolutionary rates were estimated using suitable models selected for each dataset, as described above. Statistical analyses were performed using the Kruskal–Wallis *t*-test and Horm’s method for EZR [27]. The *p*-value of less than 0.05 was considered statistically significant. Details of the phylogenetic tree analyses are shown in Table 1.

### 2.3. Phylogenetic Distance Analyses

To calculate the phylogenetic distance among the present strains, we used the ML method by MEGA7 software [28]. Also, to estimate the best substitution models, we used the Bayesian information criterion (BIC) by jModelTest2. The phylogenetic distance between the present strains were calculated with a maximum likelihood (ML) tree by MEGA7 software and estimated by the Patristic program [29]. Statistical analyses were performed using the Kruskal–Wallis *t*-test and Horm’s method for EZR. The *p*-value of less than 0.05 was considered statistically significant.

### 2.4. Phylodynamic Analyses

To evaluate the phylodynamics of the present strains, the effective population sizes of the *stx2A* gene were estimated by Bayesian skyline plot (BSP) analysis; we used the BEAST package [30]. The best models were estimated as described above. The BSP and the 95% highest probability density (HPD) were visualized by Tracer. Details of the BSP analyses are shown in Table 1.

### 2.5. Selective Pressure Analyses

To estimate sites under positive or negative selection in the Stx2A protein of the present strains, we calculated the values of the non-synonymous (*d*N) and synonymous (*d*S) at every codon position with the Datamonkey package (https://www.datamonkey.org/; accessed on 15 January 2024) [31]. We performed the fixed effects likelihood (FEL), the internal fixed effects likelihood (IFEL), the single likelihood ancestor (SLAC), and the fast unconstrained Bayesian approximation (FUBAR) methods. The selection of positive (*d*N/*d*S > 1) and negative (*d*N/*d*S < 1) selection was based on *p* < 0.05 for SLAC, FEL, and IFEL, and posterior probabilities > 0.9 for FUBAR.

### 2.6. Construction of the 3D Structure of Stx2A

To evaluate the relationship of the amino acid substitutions among the present reference strains, we constructed the three-dimensional structural models of the Stx2A protein for each subtype (Stx2a−Stx2h and Stx2o) using the homology modeling. The 1R4P (PDB ID) was selected as the template for Stx2a, 2b, 2g, 2h, and 2o, and 7U6V for Stx2c and 2d, 4P2C for Stx2e, and 6U3U for Stx2f protein, respectively, based on the amino acid sequence identity. These PDB and FASTA files were downloaded from PDB (last accessed on 4 January 2024). These amino acid sequences were aligned by MAFFTash and the models were constructed with MODELER v.10.4 [32]. The optimal models for each subtype were selected based on Ramachandran plot analysis in WinCoot v.0.9.4.1 [33]. The best models were energetically minimized using GROMOS96 in the Swiss–PDB viewer software v4.1.0 [34].

### 2.7. Conformational B-Cell Epitope Prediction

To assess the conformational B-cell epitopes of the constructed Stx2A protein models, we used the SEPPA 3.0 method (http://www.badd-cao.net/seppa3/; accessed on 1 February 2024) with a cutoff value of 0.064 [35]. Regions with contiguous amino acid sequences predicted by SEPPA 3.0 to have more than three residues were considered conformational epitopes. These epitopes were then mapped onto the constructed Stx2A protein models.

## 3. Results

### 3.1. Time-Scaled Phylogeny

**Figure 1:**
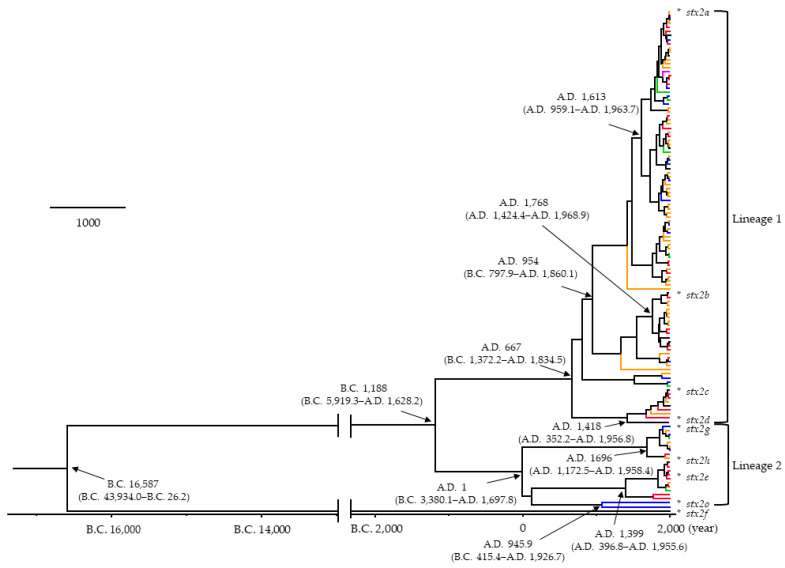
Time-scaled phylogenetic tree of the present strains in *stx2A* gene using the Bayesian MCMC method. The divergence times with 95% highest probability densities (95%HPDs) are shown in the phylogenetic tree. The asterisk (*) indicates the reference strains for each subtype. The regions were color-coded as follows: North America in blue, South America in green, Europe in red, Asia in orange, and Oceania in purple. The common ancestor of the present strains is estimated to date back about 18,600 years, subsequently diverged into *stx2a*-*2e*, *2g*, *2h*, *2o*, and *stx2f* subtypes. Statistical analyses were performed using the Kruskal–Wallis *t*-test and Horm’s method. The evolution rates of the strains belonging to Lineage 2 were statistically higher than those of the strains in Lineage 1 (*p* < 0.001).

To estimate the phylogenetic evolution of the *stx2A* gene in *E. coli* isolated from humans, animals, and foods, we constructed a time-scaled phylogenetic tree based on the MCMC method (Figure 1). Firstly, the common ancestor of the present strains was estimated to date back to around 18,600 years ago (B.C. 16587; 95% HPDs, B.C. 43934.0–B.C. 26.2). Afterward, the *stx2A* gene diverged into the *stx2a*–*2e*, *2g*, *2h*, and *2o* subtypes, and the *stx2f* subtype. Furthermore, the common ancestor between the *stx2a*–*2d* subtypes (Lineage 1) and the *stx2e*, *2g*, *2h*, and *2o* subtypes (Lineage 2) was estimated to date back to around 3200 years ago (B.C. 1188; 95%HPDs, B.C. 5919.3–A.D. 1628.2). The strains belonging to Lineage 1 further diverged and formed the *stx2a*–*2d* subtypes over approximately 1350 years, while other subtypes diverged and formed the *stx2e*, *2g*, *2h*, and *2o* subtypes over approximately 2020 years. Each *stx2A* subtype was widely distributed geographically. Next, an evolutionary rate of all strains was estimated as 8.42 × 10^–5^ substitutions/site/year (s/s/y). Interestingly, the evolution rates of the strains belonging to Lineage 2 (21 strains) were statistically higher than those of the strains in Lineage 1 (103 strains; 6.44 × 10^–5^ s/s/y vs. 5.01 × 10^–5^ s/s/y; *p* < 0.001). Furthermore, there was no distinct correlation between each strain and the countries where these were detected (Table S1). These results suggest that the *stx2A* genes evolved and diverged over an extremely long time, resulting in many subtypes.

### 3.2. Phylogenetic Distances

**Figure 2:**
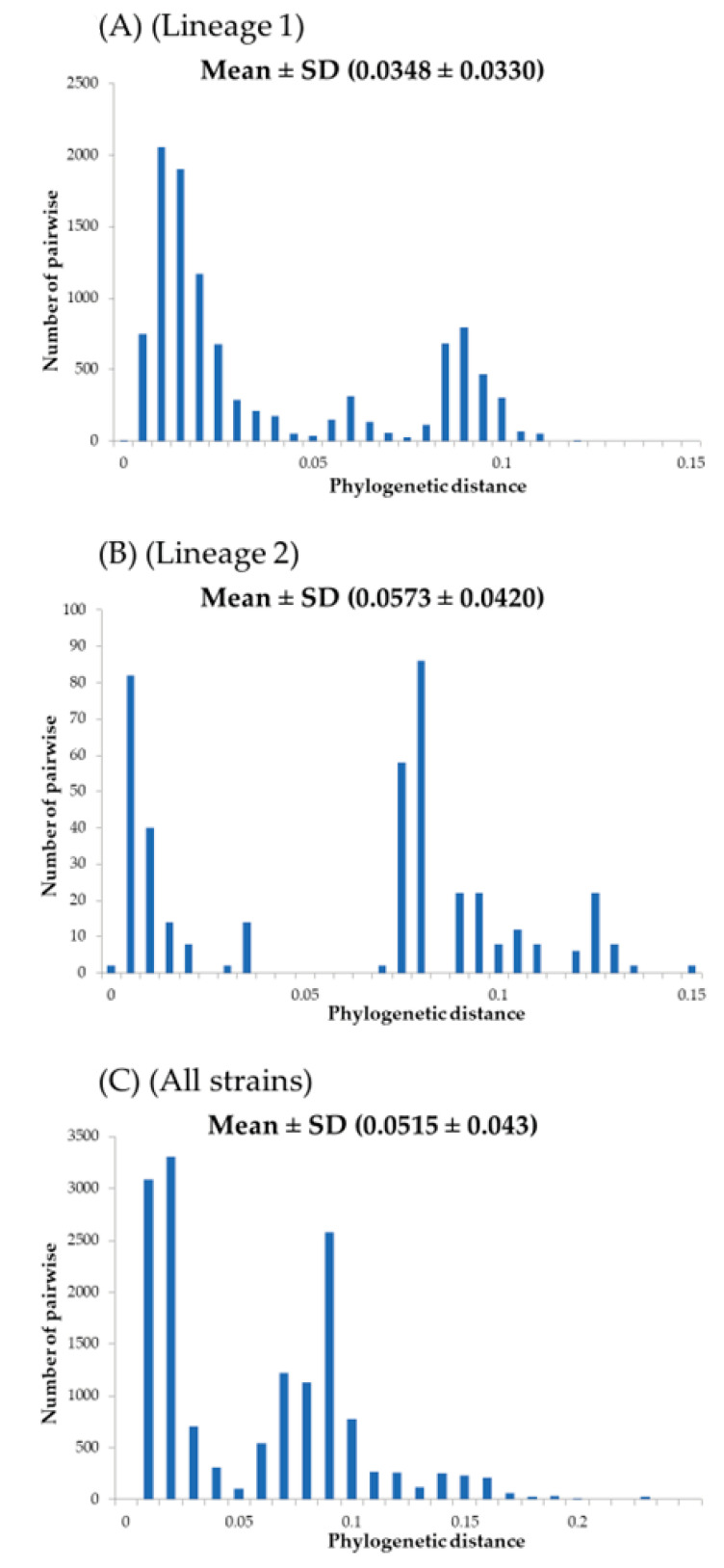
Phylogenetic distance of the (**A**) Lineage 1; (**B**) Lineage 2; (**C**) All strains represented as bar graph. The vertical axis indicates the number of pairwise and the horizontal axis indicates the phylogenetic distance. The mean and standard deviation of the phylogenetic distances are shown in the Figure. Statistical analyses were performed using the Kruskal–Wallis *t*-test and Horm’s method. Statistical analyses showed that the phylogenetic distances of the Lineage 2 strains were wider than those of Lineage 1 strains (*p* < 0.001).

In this study, to estimate the genetic divergence of the major *stx2* subtypes (except *stx2f*), we calculated phylogenetic distances (Figure 2A, B). Firstly, phylogenetic distances of the strains belonging to Lineage 1 (*stx2a*–*2d* subtypes) ranged from 0.0348 ± 0.033 and showed a trimodal distribution, while the Lineage 2 strains (*stx2e*, *2g*, *2h*, and *2o* subtypes) ranged from 0.0573 ± 0.042 and showed a multimodal distribution. Statistical analyses showed that the phylogenetic distances of the Lineage 2 strains were wider than those of Lineage 1 strains (*p* < 0.001). These results indicate that the Lineage 2 strains containing the *stx2e*, *2g*, *2h*, and *2o* subtypes had a wider genetic divergence than the *stx2a*–*2d* subtypes.

### 3.3. Phylodynamics

**Figure 3:**
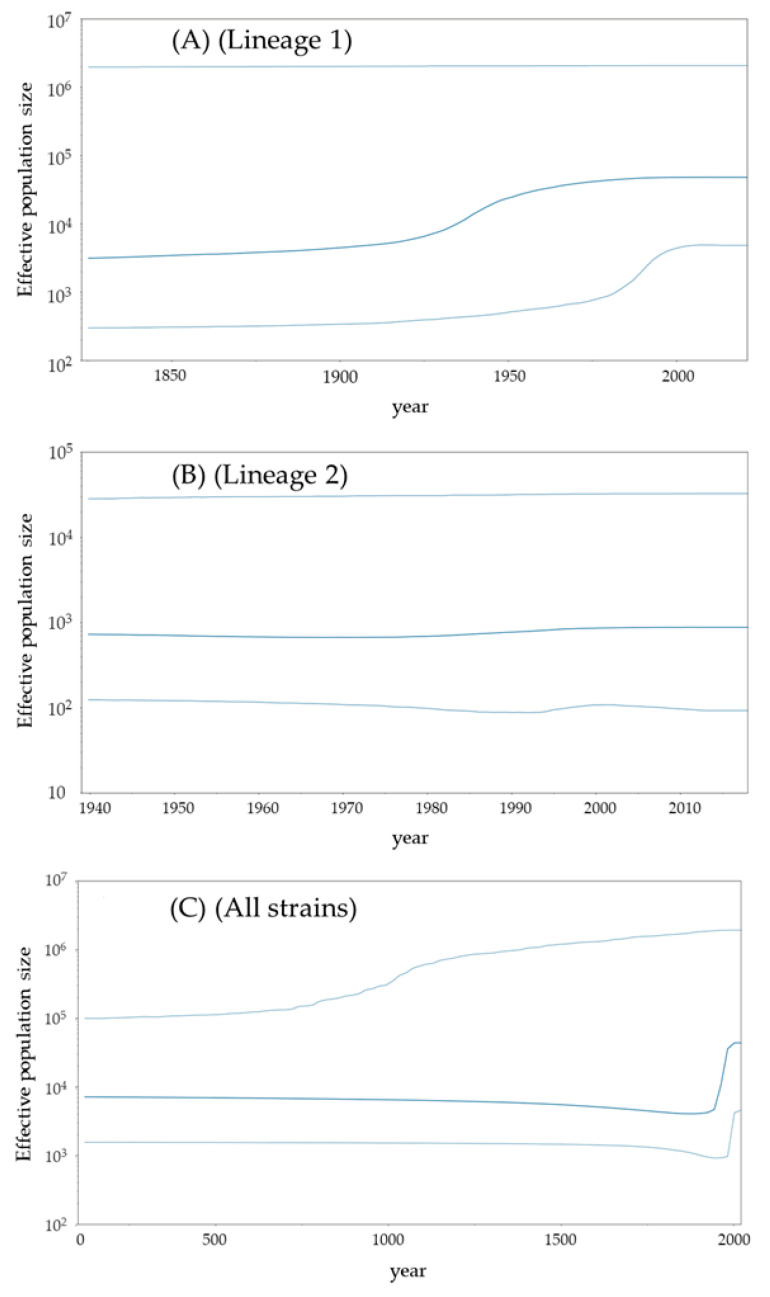
Phylodynamics of the present *stx2A* gene determined by Bayesian skyline plot analysis. (**A**) Lineage 1; (**B**) Lineage 2; (**C**) All strains. The x-axis shows time (year), and the y-axis represents the effective population size for each distance. The thick line in the center indicates the median effective population size, and the thin lines above and below show the 95% HPD. The genome population size of the Lineage 1 strains significantly increased around the 1930s. By contrast, the size of lineage 2 remained constant. Figure 3C shows the merged Lineage 1 and 2, where the upper line is not straight due to the presence of two peaks.

To estimate the past population dynamics of the pathogen, we analyzed the phylodynamics of the *stx2A* genes using the Bayesian skyline plot analysis method (Figure 3A–C). The genome population size of the Lineage 1 strains significantly increased around the 1930s (Figure 3A), while the size of Lineage 2 remained constant (Figure 3B). The size of all strains also significantly increased around the 1930s (Figure 3C). This increase may be attributed to the expansion of Lineage 1. These results suggest that Stx2-producing EHEC emerged after the 1930s and spread worldwide.

### 3.4. Relationships between Selective Pressure Sites and Active Sites

**Figure 4:**
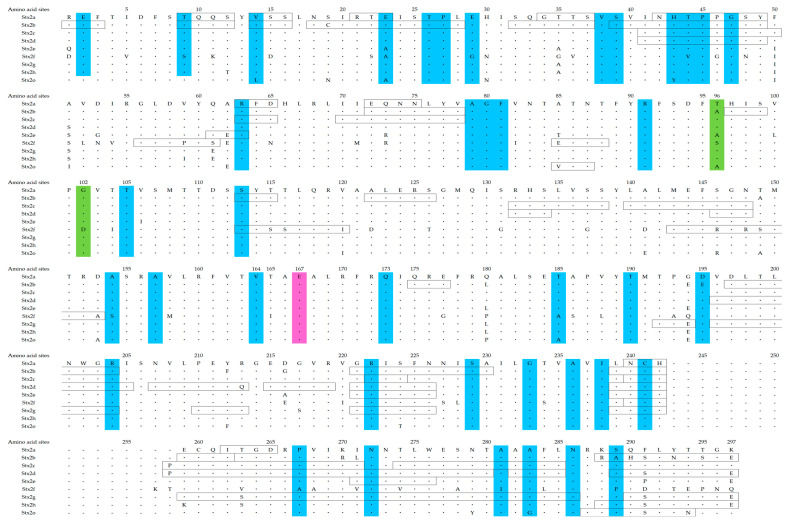
The Stx2A protein amino acid sequences investigated in this study. Amino acid sequences were aligned based on PDB data for each subtype of Stx2A and amino acid numbers were indicated. The black line boxes in the sequence are the conformational epitope. The negative selection sites, positive selection sites, and active site are highlighted in blue, green, and pink, respectively. Hyphens denote gaps not present in the PDB files. Forty negative selection sites were identified, two of which (aa164V and aa173Q) were located adjacent to the common Stx2 active site (aa167E). Only two positive selection sites were observed (aa96T or A or S, and aa102G or D).

To estimate the selective pressure against the host defense mechanisms (i.e., host immunity), we performed selective pressure analyses (Figure 4). Firstly, many negative selection sites (40 sites) were identified in all the Stx2 subtypes, and these were diffusely distributed in the Stx2A chain. Among them, two negative selection sites (aa164V and aa173Q) were located adjacent to an active site (aa167E) of the common Stx2. Only two sites of positive selection were observed (aa96T or A or S, and aa102G or D) in the protein. These sites were not nearly located at an active site. Thus, these might not be associated with the function of the protein. The results suggest that the Stx2 protein is less susceptible to positive selection pressure from the host’s immune system.

### 3.5. Conformational Epitope Analyses

**Figure 5:**
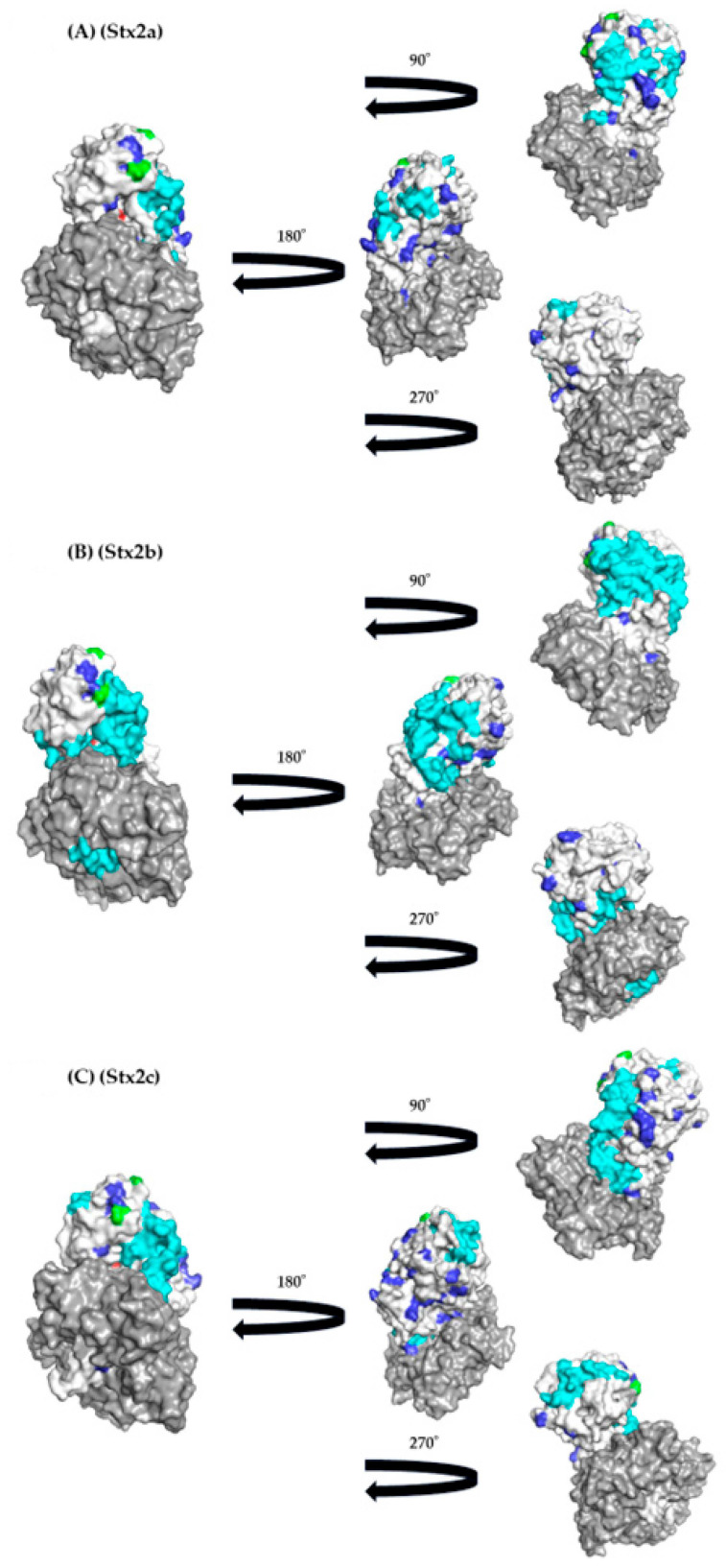

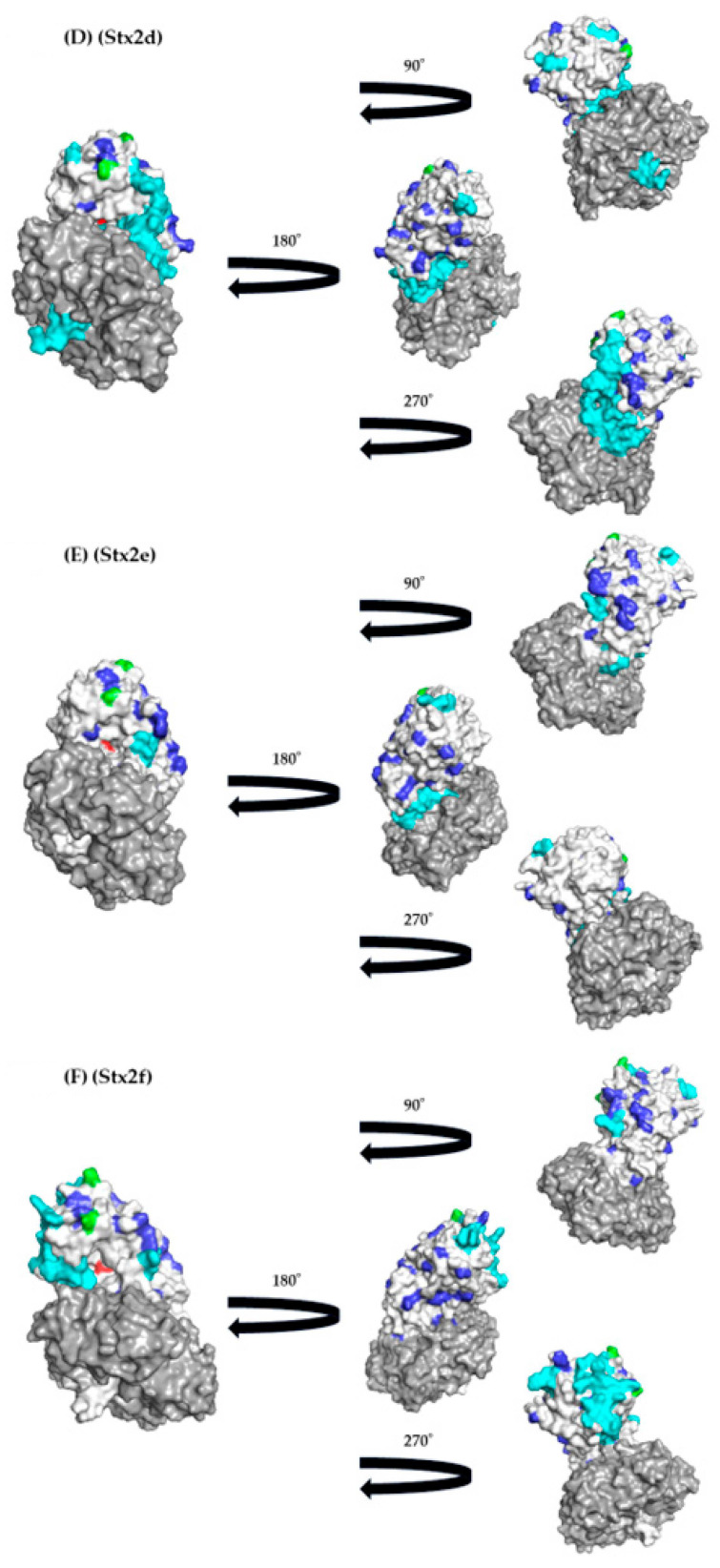

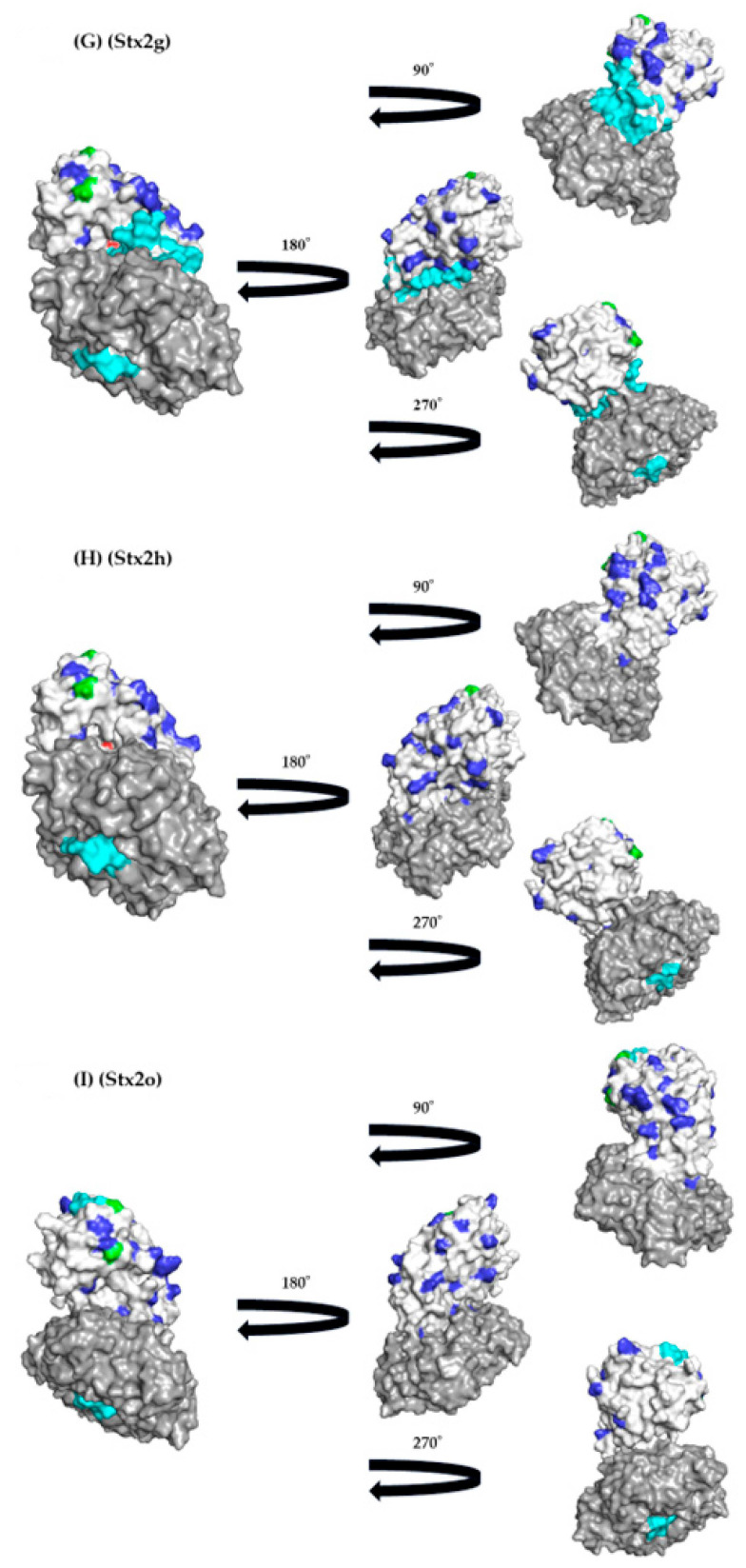
Three-dimensional (3D) structure and mapping of the Stx2A protein. Stx2A is shown in white and Stx2B structure in gray. The negative selection sites, positive selection sites, and active sites of Stx2A are indicated in blue, green, and red, respectively. Conformational epitopes are shown in cyan, and overlapping with negative selection sites are shown in priority to the conformational epitopes. Many conformational epitopes were identified for each subtype, but no common epitopes were identified among the Stx2a–2h and 2o proteins.

To gain a detailed understanding of the immunogenicity of the Stx2A proteins, we constructed three-dimensional structures and mapped conformational epitopes (Figure 5A–I). As a result, many conformational epitopes were identified in each subtype. However, a common epitope was not identified among the Stx2a–2h, and 2o proteins. Interestingly, some conformational epitopes may be located adjacent to the active site in some subtypes of the Stx2 proteins, including Stx2b–2d, 2f, and 2g (Figure 4 and Figure 5A–H). These results suggest that the host (humans) may be able to produce effective neutralizing antibodies against some subtypes of the Stx2A proteins.

## 4. Discussion

Previous reports have suggested that the *stx2A* gene shows large genetic divergence, resulting in many *stx2A* gene subtypes (*stx2a*–*2h* and *2o* subtypes), while both the *stx1* gene and the Stx1 protein appear relatively conserved [6,8,9,10]. However, the phylogeny of the *stx2A* gene may not be precisely known [8]. Therefore, we constructed a time-scaled phylogenetic tree using globally collected strains with the full length of the nucleotides of the *stx2A* genes. As a result, our findings showed that the common ancestor of the gene dated back to over 18,600 years (Figure 1). We also observed the divergence chronology of each subtype, although relationships between the subtypes of *stx2* and regional distribution were not found (Figure 1). Moreover, we estimated that the evolutionary rate of the *stx2A* gene is relatively fast (8.42 × 10^−5^ s/s/y) with wide genetic divergences (Figure 2A–C). It is well known that the *stx1* and *stx2* genes in EHEC are encoded in Stx phages (Bacteriophages) [36]. In general, evolutionary rates of the most constitutive genes range from 10^–7^ to 10^−9^ s/s/y [37,38,39]. These results suggest that the *stx2A* gene uniquely evolved with a high evolution rate, possibly influenced by the involvement of bacteriophages in the evolution of the Stx protein. To the best of our knowledge, these data may also be the first report of this.

Next, it has been thought that the Stx2-producing EHEC is an emerging bacterium [6]. However, the genetic population dynamics (phylodynamics) with the genetic flow of the bacteria may not be well understood. Thus, we performed phylodynamic analyses using Bayesian skyline plot analyses (Figure 3A–C) [30]. Interestingly, the effective population sizes (EPS) rapidly increased after the 1930s and remained constant until around 2020. A previous report showed that over 10^3^ of the EPS may explain a past epidemic [40,41,42]. In the present data, Lineage 1 including the *stx2a*–*2d* subtypes exceeded 10^4^ of the EPS, while other subtypes (*stx2e*, *2g*, *2h*, and *2o*) were less than 10^3^ of EPS. As a possible reason, the Stx2-producing EHEC was first reported in the United States [43]. In some countries, including the United States, advancements in agricultural technology during the 1930s led to the mass production of grains and the large-scale expansion of livestock farming [44]. The subsequent increase in the number of cattle may have influenced the spread and evolution of EHEC. These developments might be associated with the history of the *stx2* gene evolution. Thus, the subtypes including Lineage 1 were dominantly prevalent for over 50 years. Previous reports have suggested the biological merits of Stx production [11,12]. First, Stx may suppress the host’s immune system, allowing EHEC to evade immune responses and persist in the host [13]. Second, Stx can cause severe diarrhea, facilitating transmission to other hosts through fecal matter [14]. These factors may contribute to the EHEC’s ability to effectively spread infections [13]. Moreover, previous reports have also suggested that the virulence of Stx is distinct in each subtype [45]. In the present study, the EHEC strains belonging to Lineage 1 were dominant in the phylogenetic tree (Figure 1). It is a possibility that differences in virulence may be involved in the prosperity of the offspring [45]. These findings may also be a first.

Previous reports have shown that host defense mechanisms against bacteria including immunity, affect selective pressures, leading to positive or negative selection against the bacteria producing proteins [46,47,48]. Negative selection pressure, also known as purifying selection, refers to the evolutionary process where harmful genetic traits or mutations are removed from a population [46,47,48]. This pressure works to reduce the frequency of detrimental alleles, thereby maintaining the overall genetic integrity of the population [46,47,48]. For example, many negative selection sites are found in the cephalosporinase derived from *Pseudomonas aeruginosa* [48]. These negative selection sites may act to prevent a deterioration of the enzymatic activities [48]. In general, positive selection sites may reflect an escape from the host defense mechanisms such as cellular and humoral immunity [49]. In the present data, many negative selection sites (40 sites) were estimated in the Stx2 (Figure 4 and Figure 5). Of them, some negative selection sites (aa164V and aa173Q) were found to be adjacent to an active site of the Stx2 proteins (Figure 5). These results may act to maintain the toxicity of the Stx2. Moreover, only two positive selection sites were estimated (aa96T or A or S, and aa102G or D). As a result, the positive selection sites were not located near the active site, suggesting that the toxin protein was under less selective pressure. Thus, these sites might not be associated with this protein’s functions (Figure 4 and Figure 5). This also may be the first data regarding this.

Finally, to understand the immunogenicity of the Stx2 protein, we performed conformational epitope analyses. As a result, many conformational epitopes were estimated in the Stx2A proteins (Figure 4). Of them, some conformational epitopes may correspond to the active site in some subtypes of Stx2 proteins, including Stx2b–2d, 2f, and 2g (Figure 4 and Figure 5A–I). It was shown that the antibodies to neutralize the toxin may be able to bind to the active sites or adjacent sites [50]. Thus, human immunity may be able to produce effective neutralizing antibodies against some subtypes of the Stx2A proteins. In contrast, previous reports also suggested that some EHEC can reinfect individuals [51]. Such EHEC subtypes may be Stx2a, 2e, 2h, and 2o, although we did not examine these in vitro in this study. Next, previous reports have suggested that most of the epitopes derived from microbial proteins are conformational epitopes, while linear epitopes are few [52,53]. Thus, we analyzed conformational epitopes alone in the present study. Moreover, to predict conformational epitopes, we used an available tool (SEPPA 3.0). A previous report assessed conformational epitopes using various tools to more usefully predict conformational epitopes [53]. As a result, SEPPA 3.0 was a superior tool for the prediction of the epitopes derived from the various microbial proteins [35]. Thus, we used the tool alone in this study. Taken together, the immunogenicity of the Stx2A protein based on the present study may partially explain reinfection and the lack of changes in the pathogenicity of Stx2-producing EHEC.

## 5. Conclusions

We performed detailed molecular evolutionary analyses of the various *Shiga toxin type 2 subunit A* genes (*stx2a*–*2h*, and *2o* genes) using several bioinformatics technologies. As a result, the common ancestor of the *stx2A* gene was estimated to date back to around 18,600 years ago. Afterward, the *stx2A* gene diverged into two major lineages (Lineage 1 and 2). The Lineage 1 strains further diverged and formed the *stx2a*–*2d* subtypes, while then other subtypes diverged and formed the *stx2e*, *2g*, *2h*, and *2o* subtypes in Lineage 2. The evolutionary rate of all the strains was relatively fast. Additionally, the phylogenetic distance results showed that the Lineage 2 strains had a wider genetic divergence than the Lineage 1 strains. Phylodynamic analyses also suggested that the Stx2-producing EHEC emerged after the 1930s and spread around the world. Furthermore, many negative selection sites were estimated in the proteins. Two negative selection sites were located adjacent to an active site of the common Stx2A protein. Moreover, many conformational epitopes were estimated in these proteins. Of them, some conformational epitopes were found to be adjacent to the active site. These results imply that the *stx2A* genes evolved and diverged over an extremely long time, resulting in many subtypes. The dominance of strains belonging to Lineage 1 in the phylogenetic analysis may be due to differences in virulence. Furthermore, the Stx2A protein may be able to induce effective neutralizing antibodies against some subtypes of the proteins in humans.

## Figures and Tables

**Table 1:** Parameters used in the Bayesian Markov chain Monte Carlo (MCMC) analyses and Bayesian skyline plot analyses in this study.

	Substitution Model	Gamma Shape	Proportion Invariant	Clock Models	Demographic Models	Chain Length	Log Every
All strains (125 strain)	HKY-Γ-I	0.398	0.304	Relaxed Clock Exponential	Coalescent Constant Population	250,000,000	10,000
All strains (124 strains except *stx2f*)	HKY-Γ-I	0.398	0.304	Relaxed Clock Exponential	Coalescent Bayesian Skyline	250,000,000	10,000
Lineage 1 (103 strains)	TPM2uf-Γ-I	0.741	0.724	Relaxed Clock Exponential	Coalescent Bayesian Skyline	250,000,000	10,000
Lineage 2 (21 strains)	HKY-Γ	0.104	-	Relaxed Clock Exponential	Coalescent Bayesian Skyline	250,000,000	10,000

## Data Availability

The data presented in this study are available on request from the corresponding author.

## Supplementary Materials

The following supporting information can be downloaded at: https://www.mdpi.com/article/10.3390/microorganisms12091812/s1, Table S1: Detailed data on the strains used in this study.
