# Supplementary material for: Molecular Evolutionary Analyses of Shiga toxin type 2 subunit A Gene in the Enterohemorrhagic Escherichia coli (EHEC)
Source: Microorganisms. 2024 Sep 2;12(9):1812. doi: 10.3390/microorganisms12091812 (PMC11434168; doi:10.3390/microorganisms12091812)
Supplement: Supplementary file 1 [file microorganisms-12-01812-s001.zip › microorganisms-3137345-supplementary.pdf]

Supplementary data

Molecular evolutionary analyses of *Shigatoxin type 2 subunit A* gene in the Enterohemorrhagic *Escherichia coli* (EHEC)

Ryusuke Kimura[1, 2], Hirokazu Kimura[2, 3*], Tatsuya Shirai[2, 4], Yuriko Hayashi[2], Yuka Sato-Fujimoto[5], Wataru Kamitani[6], Akihide Ryo[4], and Haruyoshi Tomita[1]

[1] Department of Bacteriology, Gunma University, Graduate School of Medicine, Gunma, Japan: [2]Advanced Medical Science Research Center, Gunma Paz University, Gunma, Japan.: [3] Department of Health Science, Gunma Paz University Graduate School of Health Sciences, Gunma, Japan: [4]Department of Virology III, Infectious Disease Surveillance Center, National Institute of Infectious Diseases, Tokyo, Japan: [5]Faculty of Healthcare, Tokyo Healthcare University, Tokyo, Japan: [6]Department of Infectious Diseases and Host Defense, Gunma University Graduate School of Medicine, Gunma, Japan.

Email: h-kimura@paz.ac.jp

Table S1 Detailed data on the strains used in this study.

GenBank Accession Number	Host or Origin	Collection date (year)	Country	Remarks
AB015057	Musca domestica	1997	No Data (ND)	*stx2c* reference strain
AB048222	Wild deer	1997	Japan	
AB048223	Wild deer	1997	Japan	
AB048226	Wild deer	1997	Japan	
AB048227	Wild deer	1998	Japan	*stx2h* reference strain
AB048228	Wild deer	1998	Japan	
AB048229	Wild deer	1998	Japan	
AB048230	Wild deer	1998	Japan	
AB048236	Sheep	1997	Japan	
AB048238	Sheep	1997	Japan	
AB071845	Bovine	1996	Japan	
AB854278	Human	2007	Japan	
AF043627	Human	1995	ND	*stx2b* reference strain
AJ272135	Human	1997	Germany	
AJ313015	Human	1999	ND	
AJ313016	Human	1998	ND	
AJ567996	Human	1998	Germany	
AJ567997	Human	1998	Germany	
AJ567998	Human	1997	Germany	*stx2e* reference strain
AP024924	Human	2016	Japan	
AP026739	Human	2007	Japan	*stx2a* reference strain
BFKM01000027	Bovine	2014	Japan	
BFKT01000144	Bovine	2014	Japan	
BFKU01000378	Bovine	2014	Japan	
BFKW01000030	Bovine	2014	Japan	
BFXB01000097	Bovine	2014	Japan	
BFXE01000024	Bovine	2014	Japan	
BFXF01000057	Bovine	2014	Japan	
BFXO01000047	Bovine	2014	Japan	
BFXZ01000151	Bovine	2014	Japan	
BFYA01000091	Bovine	2014	Japan	
BFYB01000049	Bovine	2014	Japan	
BFZE01000029	Bovine	2013	Japan	
BGAG01000027	Bovine	2013	Japan	
BGAL01000003	Bovine	2013	Japan	
BGAQ01000040	Bovine	2013	Japan	
BGAV01000061	Bovine	2013	Japan	
BGDU01000077	Bovine	2014	Japan	
BGDW01000072	Bovine	2014	Japan	
BGDZ01000022	Bovine	2014	Japan	

BGEJ01000076	Bovine	2014	Japan	
BGFV01000083	Bovine	2014	Japan	
BGFY01000099	Bovine	2014	Japan	
BGFZ01000056	Bovine	2014	Japan	
BGGB01000036	Bovine	2014	Japan	
BGGJ01000240	Bovine	2014	Japan	
BGIA01000167	Bovine	2014	Japan	
BGIS01000032	Bovine	2014	Japan	
BHZO01000050	Bovine	2018	USA	
BIED01000052	Bovine	2018	France	
BJQW01000038	Human	2014	Japan	
BJRP01000100	Human	2014	Japan	
CP003297	Human	2009	Georgia	
CP009104	Bovine	2009	USA	
CP015240	Human	2011	USA	
CP015244	Pig	2008	USA	
CP016628	Bulgogi	2013	South Korea	
CP023061	Human	2016	South Korea	
CP023200	Buffalo	2016	India	
CP024997	Human	2017	China	
CP027328	Human	2013	ND	
CP027368	Human	2014	ND	
CP027452	Human	2013	ND	
CP027591	Human	2013	ND	
CP039404	Human	2017	ND	*stx2f* reference strain
CP043478	Yak	2016	China	
CP045209	Ground beef	2005	USA	
CP045213	Human	2002	Australia	
CP062744	Bovine	2014	United Kingdom	
CP073593	Human	2018	United Kingdom	
CP101292	Human	2021	ND	
CP110094	Bovine	2019	Turkey	
CP110095	Bovine	2019	Turkey	
DQ059012	Human	2002	Denmark	*stx2d* reference strain
DQ344636	Human	2001	Denmark	
EF441605	Food	1994	ND	
EF441622	Bovine	1990	Canada	
FM998838	Minced meat	2007	Germany	
FM998840	Ground beef	2007	Germany	
FM998842	Ground mixed meat	2007	Germany	
FM998844	German raw sausage	2007	Germany	
FM998846	Ground pork	2008	Germany	
FM998854	Ground mixed meat	2008	Germany	
FM998861	Ground beef	2007	Germany	

FN182284	Ground pork	2008	Germany	
FN252457	Bovine milk	2005	Germany	
FR850031	Rabbit meat	2009	Germany	
GQ429170	Ground beef	2006	ND	
GU126552	Human	2003	Germany	
JQ411011	Bovine	2011	Indonesia	
KF932368	Molothrus ater	2009	USA	
KF932371	Bovine	2008	USA	
KF932378	Bovine	2009	USA	*stx2g* reference strain
KU158856	Ochotona curzoniae	2013	China	
KU158857	Ochotona curzoniae	2015	China	
KU158858	Ochotona curzoniae	2015	China	
KU158859	Ochotona curzoniae	2015	China	
KU158861	Ochotona curzoniae	2015	China	
LM995896	Human	2009	Norway	
LM996832	Human	2010	Norway	
LM996947	Human	2010	Norway	
LM997161	Human	2010	Norway	
MRVS01000020	Human	2016	Israel	Reply 1
MZ229604	Human	2003	Canada	*stx2o* reference strain
MZ229605	Flour	2017	Canada	
MZ229606	Sprouts	2016	Canada	
MZ229607	Flour	2017	Canada	
PIOQ01000011	Bovine	2016	USA	
PQTM01000002	Human	2016	Bolivia	
QAFC01000029	Beef	2006	USA	
QAFL01000080	Beef	2006	USA	
QERW01000067	Human	2007	Spain	
QESD01000061	Bovine	2010	Argentina	
QESF01000075	Bovine	2009	Argentina	
QESG01000058	Bovine	1998	Argentina	
QESH01000096	Bovine	2008	Argentina	
QESN01000037	Bovine	2009	Argentina	
QESQ01000086	Bovine	2003	Argentina	
QESR01000059	Bovine	2003	Chile	
QFSP01000094	Human	2013	Czech Republic	
QRFK01000037	Human	2017	Sweden	
QRFN01000047	Human	2005	Sweden	
VZEL01000062	Human	2017	USA	
VZGK01000088	Human	2018	USA	
VZIA01000052	Human	2018	USA	
